# Modulation of HMGB1 Release in APAP-Induced Liver Injury: A Possible Strategy of Chikusetsusaponin V Targeting NETs Formation

**DOI:** 10.3389/fphar.2021.723881

**Published:** 2021-07-21

**Authors:** Jian Liu, Min Jiang, Quan Jin, Yan-Ling Wu, Zhen-Yu Cui, Ben-Wen Cui, Yue Shang, Zi-Ying Zhan, Yong-Ce Lin, Jing-Ya Jiao, Mei-Hua Piao, Zhi-Hong Zhang, Rong-Hui Sun, Ji-Xing Nan, Li-Hua Lian

**Affiliations:** ^1^ Key Laboratory of Traditional Chinese Korean Medicine Research (Yanbian University), State Ethnic Affairs Commission, Yanji, China; ^2^ Key Laboratory of Natural Medicines of the Changbai Mountain, Ministry of Education, College of Pharmacy, Yanbian University, Yanji, China; ^3^ Key Laboratory for Traditional Chinese Korean Medicine of Jilin Province, College of Pharmacy, Yanbian University, Yanji, China; ^4^ Clinical Research Centre, Yanbian University Hospital, Yanji, China

**Keywords:** acetaminophen, drug-induced liver injury, HMGB1, neutrophil extracellular traps, chikusetsusaponin V

## Abstract

Acetaminophen (APAP), one of the most common antipyretic analgesics, which is safe at therapeutic dose, cause acute liver injury and even death at overdose. However, the mechanism of APAP-induced inflammation in liver injury is still controversial. Therefore, effective drug intervention is urgently needed. The aim of this study was to explore the inflammatory exact mechanism of APAP, especially on neutrophils, and to study the intervention effect of *Chikusetsusaponin V* (CKV) derived from *Panax japonicus*. Establishment of hepatotoxicity model of APAP *in vitro* and *in vivo*. *In vitro*, HepG2 cells, AML12 cells, primary mouse hepatocytes and neutrophils were used to mimic APAP-affected hepatocytes and neutrophil. *In vivo*, C57BL/6 mice were administrated overdose of APAP with or without neutrophil depletion or abolishing neutrophil extracellular traps (NETs) formation. In this study, APAP stimulation increased the level of HMGB1, IL-1β and Caspase-1 in mouse liver, especially hepatocytes, which had a synergistic effect with LPS/ATP combination. NETs were formatted at early stage of APAP or HMGB1-stimulated neutrophils’ damage. Conditioned mediums from APAP-treated hepatocytes induced more significant NETs than direct APAP stimulation. Neutrophil depletion or abolishing NETs formation decreased HMGB1 level, eventually blocked hepatocytes necrosis. CKV pretreatment interfered Caspase-1 activation and HMGB1 release in APAP-damaged hepatocytes. CKV also prevented NETs formation. These results indicate that the production of HMGB1 may depend on the activation of Caspase-1 and play a key role in liver inflammation caused by APAP. The cross-dialogue between hepatocytes and neutrophils can be mediated by HMGB1. Therefore, CKV has a positive intervention effect on NETs-related inflammation in APAP-damaged liver, targeting Caspase-1-HMGB1.

## Introduction

Drug-induced liver disease induced by drug abuse has become the primary cause of acute liver failure and liver transplantation (Stine and Chalasani, 2015). Under the situation of the spread of the epidemic situation of COVID-19, the risk of drug-induced liver injury induced by misadministration of Acetaminophen (PubChem CID: 1983, APAP) may increase. APAP belongs to acetaniline non-steroidal antipyretic analgesics, which is one of the most commonly used antipyretic analgesics in clinic. It is safe within the therapeutic dose, but long-term, overdose or drinking during medication will increase the risk of hepatotoxicity caused by APAP (Lee, 2017; Chayanupatkul and Schiano, 2020). N-Acetyl Cysteine (PubChem CID: 12035, NAC) is the only clinical antagonist for APAP poisoning at present, but its treatment window is narrow and can only achieve sufficient curative effect within 12 h (Celona et al., 2011). Therefore, there is an urgent need to clarify the pathological mechanism of liver injury caused by APAP and to develop new therapeutic targets for its clinical treatment.

The pathological mechanism of hepatotoxicity caused by excessive APAP has obvious stages, that is, metabolic stage, injury stage and repair and regeneration stage (Mossanen et al., 2016; Woolbright and Jaeschke, 2017; Yan et al., 2018). In the injury stage of APAP hepatotoxicity, the landmark pathological phenomenon is the oxidative stress of organelles caused by mitochondrial protein conjugates, and the downstream event is the inflammatory response after cell death (Antoniades et al., 2012). Cell death and rupture release Damage-Associated Molecular Patterns (DAMPs), they are a kind of endogenous molecules released by self-cell death and play the role of endogenous danger signals. These endogenous molecules include High Mobility Group Box-1 Protein (HMGB1), DNA fragments and Adenosine Triphosphate (ATP) (Canesin et al., 2020). Among them, HMGB1 is passively released from the nucleus and binds to Toll-like Receptor 4 (TLR4) and advanced glycation end product receptor Receptor for Advanced Glycation Endproducts (RAGE) to produce related inflammatory factors and participate in the regulation of inflammation (Maroso et al., 2011). Therefore, as an important initiating factor of inflammation, DAMPs is the key node in the study of liver inflammation induced by APAP.

In addition, HMGB1 inhibitors also showed a longer treatment window than NAC (Lundbäck et al., 2016). Therefore, the development of therapeutic agents to shorten the injury phase for inflammatory targets by HMGB1 is a potential clinical strategy to improve liver injury caused by APAP. Interleukin-1β (IL-1β) is a powerful inflammatory factor, mainly produced by macrophages. Activation of Caspase-1 driven by NOD Like Receptors Protein P3 (NLRP3) inflammatory bodies promotes the release of HMGB1 and proinflammatory cytokine IL-1β through pyroptosis (Lamkanfi et al., 2010; Jiang et al., 2017; Li et al., 2018; Jiang et al., 2020). In the current study, we also explored the relationship between Caspase-1 and HMGB1 in the mechanism of liver inflammation in APAP *in vivo* and *in vitro*.

Inflammation is a common characteristic of acute and chronic liver disease caused by pathogenic factors such as drugs (Zou et al., 2009), alcohol (Mandrekar and Szabo, 2009) and viruses (Abe et al., 2010). Neutrophil extracellular traps (NETs) are large extracellular reticular structures, composed of cytoplasm and granule proteins, and assembled on a non-concentrated chromatin scaffold (Muñoz et al., 2016). Early studies found that NETs are composed of more than twenty proteins, including neutrophil elastase (NE) and myeloperoxidase (MPO) (Lood et al., 2016). However, the formation of NETs and the HMGB1 release in APAP-induced liver injury is still elusive. Therefore, we depleted neutrophils or degraded NETs in APAP-treated mice by DNase I to explore the possibility of targeting to the interactive dialogue between HMGB1 and NETs in APAP hepatotoxicity.

At present, the strategies of natural products to improve APAP liver injury mainly include anti-oxidation, anti-inflammation, inhibition of liver microsomal enzymes (Domitrović and Potočnjak, 2016). Panax japonicus (T.Nees) C. A. Mey, also known as also known as Bamboo ginseng (“Zhujieshen”), belongs to the Araliaceae family. *Chikusetsusaponin V* (PubChem CID: 11815492, CKV), a saponin from Panax japonicus has been reported to reduce the level of inflammatory cytokines in serum induced by lipopolysaccharide (LPS) by inhibiting nuclear factor-κB (NF-κB) and mitogen activated protein kinase (MAPK) signal pathway, and improve liver injury in mice (Dai et al., 2016). Thus, currently we aimed to discover the modulation of HMGB1 release targeting NETs formation in APAP-induced liver injury, and based on the strategy to investigate the underlying mechanism how CKV alleviate the APAP-induced liver injury.

## Materials and Methods

### Reagents

APAP, NAC, Histopaque®-1119, Histopaque®-1077 and ATP disodium salt (PubChem CID: 16218877) were purchased from Sigma Chemical Co. (St. Louis, MO, United States). CKV was obtained from the National Institute for the Control of Pharmaceutical and Biological Products (>99% purity; Beijing, China) and was dissolved in saline. Ultrapure LPS from *Escherichia coli* O111:B4 was purchased from InvivoGen (San Diego, CA, United States). The fluorescent secondary antibodies Goat anti-Rb IgG Alexa Fluor-488, anti-Rb IgG Alexa Fluor-594 and 4′, 6′-Diamidino-2-phenylindole (DAPI) were purchased from Abcam (Cambridge, MA, United States). The antibodies involved and their concentrations are as follows: anti- HMGB1 (Abcam, ab79823), anti- IL-1β (R & D Systems, AF-401-NA), anti- Caspase-1 (sc-56036, Santa Cruz, CA, United States), anti- P2x7R (Abcam, ab48871), anti- MAPLC3β (G-2) (Santa Cruz, sc-271625), anti- MAPLC3β (G-4) (Santa Cruz, sc-398822), anti- MAPLC3β (G-9) (Santa Cruz, sc-376404), anti- NE (Abcam, ab68672), anti- PR3 (Abcam, sc-74534), anti- RAGE (Santa Cruz, sc-365154), anti- F4/80 (Abcam, ab6640), anti- NLRP3 (Abcam, ab4207), anti-Myeloperoxidase (Abcam, ab208670). Horseradish peroxidase (HRP)-conjugated goat anti-mouse, goat anti-rabbit and donkey anti-goat antibodies were purchased from Abcam. PE anti-mouse Ly-6G Antibody was purchased from BioLegend (San Diego, CA, United States). The BCA Protein Assay Kit, Methylthiazolyldiphenyl—tetrazolium bromide (MTT) and DNase I were obtained from Beyotime (Nantong, Jiangsu, China). The cell culture mediums (Gibco) were all purchased from Invitrogen (Carlsbad, CA, United States).

### Animals and Models

Male C57BL/6 mice (8–10 weeks old, 20–22 g) were purchased from Changchun Yisi Laboratory Animal Technology Co., Ltd. [(SPF, SCXK [JI] 2018-0007), Jilin, China]. The mice were housed in an animal facility at Yanbian University and were fed a standard chow diet and given tap water ad libitum. All mice were maintained at a temperature of 23 ± 2°C, with a relative humidity of 55 ± 5% and 12/12-h light/dark cycles. All animal experiments carried out the National Institutes of Health guide for the care and use of Laboratory animals (NIH Publications No. 8023, revised 1978) and were approved by the Animal Research Committee of Yanbian University, China. The animals were treated humanely, and all efforts were made to minimize the animals’ suffering as well as the animal numbers. Animal studies are reported in compliance with the ARRIVE guidelines (Percie du Sert et al., 2020).

Mice were exposed to four different model protocols. 1) Establishment of a controlled feeding model of APAP-induced liver injury in mice: To explore the effect of eating factors on liver injury induced by APAP in mice. The mice were randomly divided into five groups (*n* = 6 per group), including a control group, APAP treatment 12 h fasting or no fasting group and APAP 24 h fasting or non-fasting group. The mice were treated by intragastric administration of APAP300 mg/kg for 12 h or 24 h, and their food intake was controlled respectively. The way of diet control is to start fasting 12 h before the administration of APAP and not to abstain from water. 2) Establishment of time gradient model of APAP-induced liver injury in mice under fasting condition: To explore the phase of liver injury induced by APAP in mice under fasting condition. The mice were randomly divided into four groups (*n* = 6 per group), including a control group, APAP300 mg/kg treated groups for 6, 12 and 24 h, respectively, and fasted does not prohibit water before 12 h administration. 3) Establishment of mouse model of neutrophil neutralization: To explore the effect of neutrophils on liver injury induced by APAP in mice. Mice were injected intraperitoneally with Ly6G antibody (200 μg/mouse) to neutralize neutrophils 24 h before APAP300 mg/kg treatment, and then treated with APAP for 12 h. The Ly6G antibody control group was set up, and the mouse were fasted 12 h before the administration of APAP and not to abstain from water. 4) Establishment of a mouse model of NETs depletion: To explore the effect of NETs on liver injury induced by APAP in mice. The mice were treated with APAP 300 mg/kg for 1 h and then injected with DNase I (100 mg/kg), APAP into the abdominal cavity for 12 h. Because the NETs of neutrophils is a DNA grid containing cytokines and chemokine proteins ejected from the cells during the suicidal death of neutrophils, it is digested with DNA enzyme to deplete it. In this experiment, DNase I control group was also set up, and the mouse were fasted does not prohibit water 12 h before the administration of APAP.

All mice were anesthetized with isoflurane. Blood was collected by direct cardiac puncture. The entire liver was removed, and a portion of tissue from the same lobe of the liver in each mouse was embedded in OCT Compound (Tissye-Tek®; Sakura Finetek, Torrance, CA) or 10% neutral buffered formalin for histological analysis. The rest of the tissue was snap frozen in liquid nitrogen and then stored at −80°C until analyzed.

### Isolation of Primary Mouse Hepatocytes

Primary hepatocytes were isolated from male C57BL/6 mice aged 10–12°weeks, and the whole liver was perfused through hepatic portal vein and inferior vena cava. First, blood from the liver was perfused with HBSS perfusion solution containing EDTA, and then the liver was perfused with William’s E Medium (WME) containing collagenase. The enzymatic hydrolyzed liver was treated into cell suspension, then the seed plate was eluted and purified with WME culture medium, and then adhered to the wall for 4 h. In order to ensure the viability and experimental consistency of primary hepatocytes, they should be separated and used at any time.

### Isolation of Mouse Neutrophils

The femur and cervical thigh of male C57BL/6 mice aged 10–12 weeks were separated. The bone marrow fluid was flushed out with RPMI medium containing 10% FBS, EDTA (2 mM) and HEPES (25 mM). After collecting cells by 427 × g elution, red blood cells were broken with 0.2 and 1.6% sodium chloride solution. After many times of elution, the cells were collected with ice PBS and separated with Histopaque solution. Histopaque1119 and Histopaque 1077 were slowly added to the test tube, respectively, and finally covered with PBS cell suspension, and the cells were isolated by centrifugation. Neutrophils were collected between Histopaque1119 and Histopaque 1077. Finally, it was eluted with EDTA-free medium for 3 times and used after 6 h of adhesion. Neutrophils should be isolated and used at any time because of their high activity and short life span.

### Cell Culture and Treatment

The human hepatoma cell line, HepG2 and the AML12 (alpha mouse liver 12) cell line, were generous gifts from Professor Jung Joon Lee (Korea Research Institute of Bioscience and Biotechnology, Daejeon, Korea).

HepG2 cells were cultured in DMEM supplemented with 10% fetal bovine serum (FBS), 100 units/ml penicillin G, and 100 mg/ml streptomycin at 37°C in 5% CO_2_. AML-12 cells were incubated in DMEM/F12 medium supplemented with penicillin (100 U/ml), streptomycin (100 mg/ml), 10% FBS and a mixture of insulin-transferrin-selenium (1%), dexamethasone (40 ng/ml), GlutaMAX (1%) and non-essential amino acids (NEAA) at 37°C under 5% CO_2_. Cells were maintained at a density between 1 × 10^5^ and 1 × 10^6^ cells/m. The cells were passaged by trypsinization every 2 or 3 days, and the experiments were performed using cells from the fourth to the seventh passages.

Primary mouse hepatocytes were cultured in Martrigel-plated plates and cultured in WME medium containing 7% FBS, dexamethasone, sodium pyruvate, and ITS at 37°C and 5% CO_2_. Mouse neutrophils were cultured in RPMI medium containing 10% FBS, HEPES (25 mM), Glutamix, and penicillin streptomycin. The serum-free medium was used when the drug was given.

AML12 cells, HepG2 cells and primary mouse hepatocytes were treated with different concentrations of APAP for 0–24 h. LPS combined with ATP was treated with LPS (1 μg/ml) for 4 h and then treated with ATP (3 mM) for 30 min. When treated with LPS/ATP and APAP, HepG2 cells were treated with APAP (10 mM), AML12 cells were treated with APAP (5 mM) for 24 h, and LPS/ATP was given a second hit in the last 4 h. The treatment of cross-dialogue between primary hepatocytes and neutrophils. The supernatant was collected after the primary hepatocytes were treated with APAP (5 mM) for 24 h. The supernatant of 200 × g centrifugal 5 min was treated and cultured on neutrophils for 2 h to detect the expression of NE. Neutrophils were stimulated with HMGB1 protein 100 ng/ml for 2, 4, and 8 h to detect the expression of NE.

### Measurement of Cell Viability by MTT Assay

The effect of APAP on cell survival rate was detected by MTT. The toxicity of AML12 and HepG2 of APAP with different concentration gradients was detected under the time gradient of 0–24 h. MTT was added and incubated for continuous 3 h. The content of purple formazan was detected using a microplate reader at 540 nm.

### Biochemical Assays

Serum alanine aminotransferase (ALT) and aspartate aminotransferase (AST) levels were detected by using a commercial colorimetric kit (Nanjing Jiancheng Bioengineering Institute, Nanjing, China) according to the manufacturer’s protocols, and then measured by Autodry Chemistry Analyzer (SPOTCHEM™ SP4410, Arkray, Japan).

### ELISA

Interleukin-1β (IL-1β) protein level was measured by mouse IL-1β/IL-1F2 DuoSet ELISA Kit from R&D Systems (DY401, Minneapolis, MN, United States) according to the manufacturers’ instructions.

### Liver Histopathological Analysis

For hematoxylin and eosin (H&E) staining, the liver sections were fixed in 10% neutral buffered formalin, dehydrated in descending grades of ethanol, and embedded in paraffin. Sections (5 μm) of the liver were deparaffinized and stained with hematoxylin and eosin in sequence. The stained slides were visualized with a Nikon TI-E fluorescence microscope (Nikon, Tokyo, Japan). All analysis was carried out in a blinded manner.

Liver apoptosis was performed with Colorimetric TUNEL Apoptosis Assay Kit purchased from Beyotime Biotechnology (C1091, Beijing, China) according to the manufacturers’ instructions. Paraffin sections (5 μm) were dewaxing and retrieval with DNase free proteinase K, blocking with Peroxidase Streptavidin after incubating slides with staining solution, HRP labeled samples were visualized with DAB. Images were acquired by BX53 upright microscope (Olympus, Tokyo, Japan). All these examinations were carried out in a blinded manner.

### Immunohistochemistry

Paraffin sections (5 μm) were deparaffinized in xylene and dehydrated in progressively increased concentration of ethanol. Then the sections were microwaved in 10 mM sodium citrate buffer (pH 6.0) and cooled to room temperature. Blocking the slides with 3% hydrogen peroxide, 5% goat serum and Streptavidin Blocking solution (SP-2002, Vector Laboratories, Inc., Burlingame, CA, United States). The sections were incubated with the primary antibodies and followed by the specific secondary antibodies. After blocking the slides with Peroxidase Streptavidin (SA-5704, Vector Laboratories, Inc., Burlingame, CA, United States), bound antibodies were visualized with Lab Vision™ DAB Plus Substrate Staining System (TA-060-HDX, Thermo Fisher Scientific, Fremont, CA, United States). Images were acquired by Ti-E inverted microscope (Nikon, Tokyo, Japan) or BX53 upright microscope (Olympus, Tokyo, Japan). All these examinations were carried out in a blinded manner.

### Single/Double Immunofluorescence Staining

The cells were fixed with 4% paraformaldehyde solution and were incubated in 0.5% Triton X-100 (v/v) in PBS for 20 min. Five-micrometre cryostat sections were fixed in a 1:1 acetone–methanol mixture. Frozen sections or cells were incubated in a blocking solution with 5% goat serum, followed by incubation with primary antibodies overnight at 4°C and then with appropriate secondary antibodies. Caspase-1 was analyzed by FLICA fluorescence staining, frozen sections were fixed and sealed with goat serum, then FLICA staining solution and washing solution were added successively to elute the unstained dye. Mounted the slides with DAPI and protected them away from light 30 min before observation. The fluorescence was visualized by a FV10i Confocal Laser Scanning Microscope from Olympus or BX53 Olympus microscope. All these examinations were carried out in a blinded manner. The immunofluorescence intensity was analyzed with Image Pro-Plus 6.0 (Media Cybernetics, Inc., MD, United States). The averaged percentage of immunostaining area was calculated from at least three nonadjacent sections of each sample.

### Western Blotting

The total proteins of cells and liver samples were extracted using lysis buffer (Abcam, Cambridge, MA, United States) supplemented with protease and phosphatase inhibitors (Roche Diagnostics, Mannheim, Germany). Equal quantities of protein (20 μg) were loaded onto an 8–12% gel, separated by SDS-PAGE, and transferred to PVDF membranes (Amersham Hybond, GE Healthcare Bio-Sciences, Pittsburgh, PA, United States). Next, the membranes were blocked with 5% skim milk in PBS containing 0.05% Tween 20 and were incubated at 4°C overnight with the primary antibodies. Thereafter, the membranes were incubated with the appropriate HRP-conjugated secondary antibodies, and the signals were visualized using the Amersham ECL Prime Western Blotting Detection Reagent (RPN2232, GE Healthcare, United Kingdom). The intensity of the protein bands was quantified using Quantity One software (Bio- Rad, CA, United States). Intensity values of the relative protein levels were normalized to GAPDH (Abcam, ab8245; 1:10000) or β-actin (Abcam, ab133626, 1:10000).

### RNA Extraction and RT-PCR

Total RNA from cells and mouse liver was isolated using Eastep® Super Total RNA Extraction Kit (LS1040, Promega, Madison, WI, United States) according to the manufacturer’s instructions. RT-PCR was performed using primers specific for the genes, primer sequences are listed in the Table 1. In brief, 1 μL of the cDNA obtained from the reverse transcription reactions were amplified in a total volume of 20 μL consisting of 1 × GoTaq reaction buffer, 2 U GoTaq DNA polymerase, 12.5 nM dNTP Mix, gene-specific primers were added at a final concentration of 200 nM (all reagents were from Promega). Thermal cycles of 30 s at 95°C, 30 s at annealing temperature, 1 min at 72°C, and a final elongation step at 72°C for 10 min. The PCR products were subjected to electrophoresis on 2% agarose and stained with ethidium bromide. The transcriptional level of the same sample was normalized by GAPDH mRNA, and the quantitative analysis and presentation followed the MIQE guidelines.

**TABLE 1:** Primer sequences used for RT-PCR

Species	Gene	Accession NO.	Primer sequence 5′-3′
*Mus musculus*	*P2rx7*	NM_001038839.2	F: CGA​ATT​ATG​GCA​CCG​TCA​AGT
			R: TTC​TCC​GTC​ACC​TCT​GCT​ATG
*Mus musculus*	*Il1b*	NM_008361.3	F: GTA​CAT​CAG​CAC​CTC​ACA​AG
			R: CAC​AGG​CTC​TCT​TTG​AAC​AG
*Mus musculus*	*Casp1*	NM_009807.2	F: ACA​TCC​TTC​ATC​CTC​AGA​AAC
			R: GAT​AAT​GAG​GGC​AAG​ACG​TG
*Mus musculus*	*gapdh*	NM_008084.2	F: CTTGTGCAGTGCCAGCC
			R: GCC​CAA​TAC​GGC​CAA​ATC​C

### Quantitative Real-Time PCR Assay

qPCR was performed on an Mx3000 P qPCR System (Agilent, Germany) with SYBR Green Master Mix (Life Technologies, Carlsbad, CA). The forward (F) and reverse (R) primers of RNA are listed in the Table 2. The level of mRNA were normalized to GAPDH, and the relative fold difference was quantified using the comparative threshold cycle (ΔΔCt) method, and the quantitative analysis and presentation followed the MIQE guidelines.

**TABLE 2:** Primer sequences used for real-time qPCR.

Species	Gene	Accession NO.	Primer sequence 5′-3′
*Mus musculus*	*Il1b*	NM_008361.3	F: TCT​TTG​AAG​AAG​AGC​CCA​TCC
			R: CTA​ATG​GGA​ACG​TCA​CAC​AC
*Mus musculus*	*Cxcl1*	NM_008176.3	F: AGC​CAC​ACT​CAA​GAA​TGG​TC
			R: GCC​ATC​AGA​GCA​GTC​TGT​C
*Mus musculus*	*Cxcl2*	NM_009140.2	F: CCA​ACC​ACC​AGG​CTA​CAG​G
			R: GCG​TCA​CAC​TCA​AGC​TCT​G
*Mus musculus*	*gapdh*	NM_008084.2	F: CTTGTGCAGTGCCAGCC
			R: GCC​CAA​TAC​GGC​CAA​ATC​C

### Statistics

All values are presented as mean ± SD. The comparison of the results was evaluated by one-way analysis of variance (ANOVA) and Tukey’s multiple comparison tests. Statistically significant differences between groups were defined as *p*-values less than 0.05. Calculations were performed using the GraphPad Prism program (Graphpad Software, Inc., San Diego, CA, United States).

## Result

### Acetaminophen Promoted Inflammatory Factors in Hepatocyte

**FIGURE 1:**
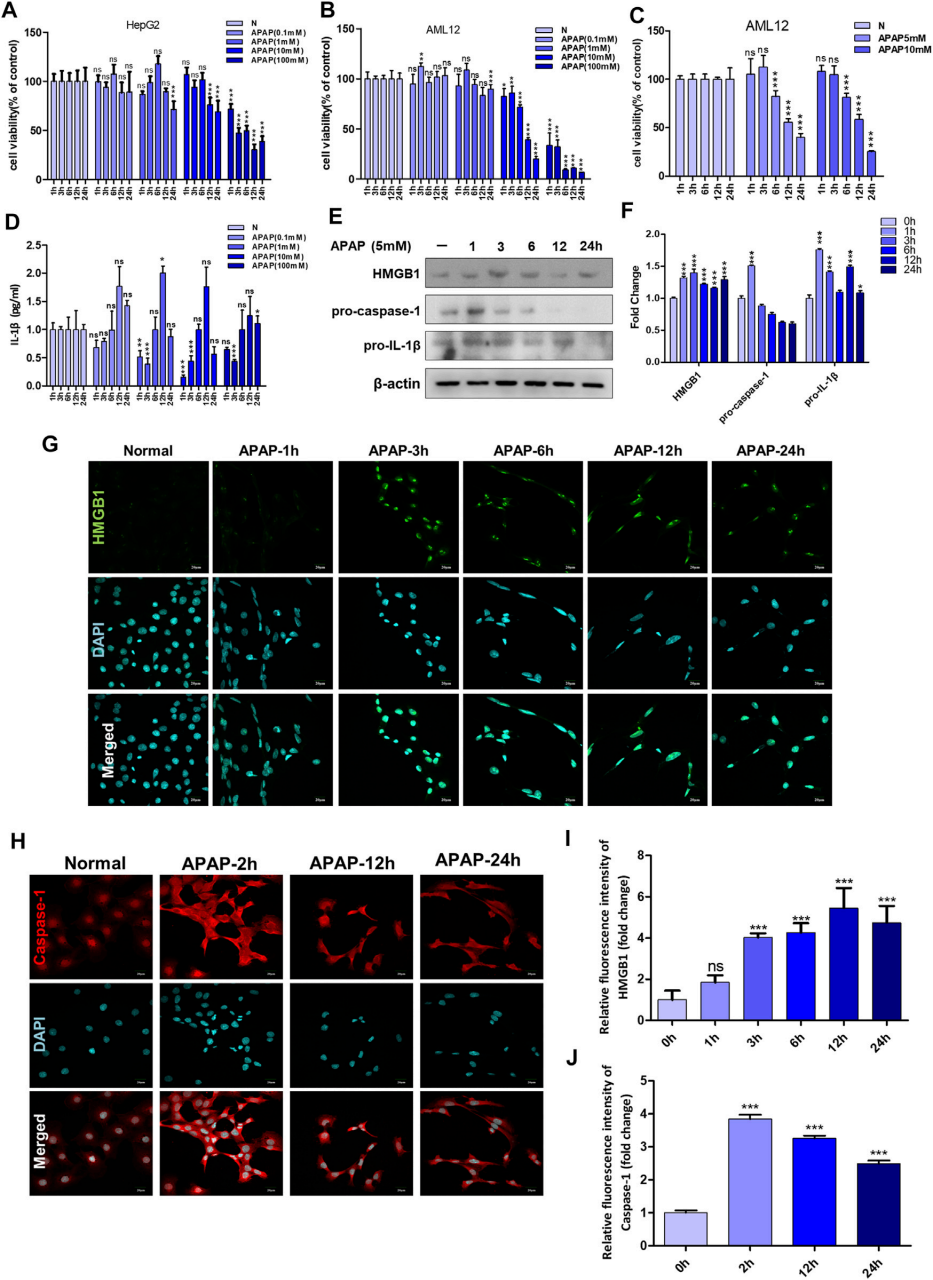
APAP *in vitro* experimental concentration screening and the influence of inflammation-related indicators after stimulating hepatocytes. **(A,B)** The effect of APAP with a concentration gradient of 0–100 mM on the cytotoxicity of HepG2 and AML12 under a 24 h time gradient. **(C)** The cytotoxicity of AML12 cells treated with a time gradient of 1–24 h at concentrations of APAP5 mM and 10 mM. **(D)** The level of IL-1β in the supernatant of AML12 cells treated with 0–100 mM APAP for 1–24 h *p < 0.05, **p < 0.01, ***p < 0.001, significantly different from 1 h group. **(E,F)** Treat AML12 cells with APAP (5 mM) under a time gradient of 0–24 h. The protein expression levels of HMGB1, pro-caspase-1 and pro-IL-1β and the gray density relative to β-actin were analyzed. **(G,H)**Fluorescence expression of HMGB1 and Caspase-1 after APAP (5 mM) stimulated AML12 cells under different time gradients within 0–24 h **(I,J)** Quantitative analysis of relative fluorescence intensity of G and H. All images were magnified by 400×. *p < 0.05, **p < 0.01, ***p < 0.001, significantly different from the normal group. One-way ANOVA followed by Tukey’s test. The data are expressed as the mean ± SD of five independent assays with at least three replicates. Green is HMGB1 staining, red is Caspase-1 staining, blue is DAPI.

Different cell lines have different tolerance to APAP, the effect of APAP on cell viability was detected in HepG2 cells and AML12 cells, with concentration gradient of 0.1–100 mM within 24 h. The results showed that when the dose of APAP given to HepG2 cells was 10 mM, the toxicity occurred in 12 h, and the toxicity occurred in 1 h when the dose was 100 mM. However, when APAP doses of 10 mM or 100 mM were given to AML12 cells, obvious cytotoxicity appeared in only 1 h. This indicates that AML12 cells are more sensitive to APAP (Figures 1A,B). Figure 1C shows the time gradient screening of AML12 cells at 5 and 10 mM doses of APAP. The results showed that under the same treatment time of 6 and 12 h, APAP 5 and 10 mM produced equivalent toxicity. As a result, it was determined that AML12 cells were used in the subsequent *in vitro* experiment, and the concentration of APAP was 5 mM.

AML12 cells were stimulated with APAP at different time gradients or concentration gradients. The results in Figure 1D showed that 0.1, 1 and 10 mM APAP began to secrete IL-1β at 12 h, but there was no change in 100 mM APAP. It is speculated that it may be due to excessive cell damage or necrosis leading to loss of cell function. In addition, at 1 h and 3 h, 0.1 mM–100 mM APAP reduced the secretion of IL-1β. There was no effect at 6 h, and IL-1β secretion level increased at 12 h, of which 1 mM was the highest. At 24 h, IL-1β secretion decreased, and there was no significant difference compared with the normal group. The secretion of IL-1β presents different response trends with 6 h as the boundary, indicating that 6–12 h is the injury phase, and the cells are undergoing complex inflammatory reactions before and after 6 h, and 6–12 h may be the time window for cell-level treatment and rescue of damaged cells.

In order to further determine the role of inflammation in the *in vitro* model of APAP, the expression of inflammation-related proteins in AML12 cells stimulated by APAP was detected at different time gradients. The results showed that the expression of pro-caspase-1 and pro-IL-1β increased after administration of APAP1 h, and showed a downward trend after 24 h (Figure 1E). The expression of HMGB1 increased after administration of APAP3 h and decreased gradually after 24 h (Figure 1G). Caspase-1 is a prerequisite for the maturation of inflammatory cytokines. The release of HMGB1 from hepatocytes is mediated by Caspase-1 through inflammatory bodies. After 2 h of cell administration, Caspase-1 responded rapidly, and its protein expression reached a peak, and gradually decreased after 24 h (Figure 1H).

### Acetaminophen Synergized With LPS/ATP Magnified Caspase-1-HMGB1 Related Inflammation in Hepatocytes

**FIGURE 2:**
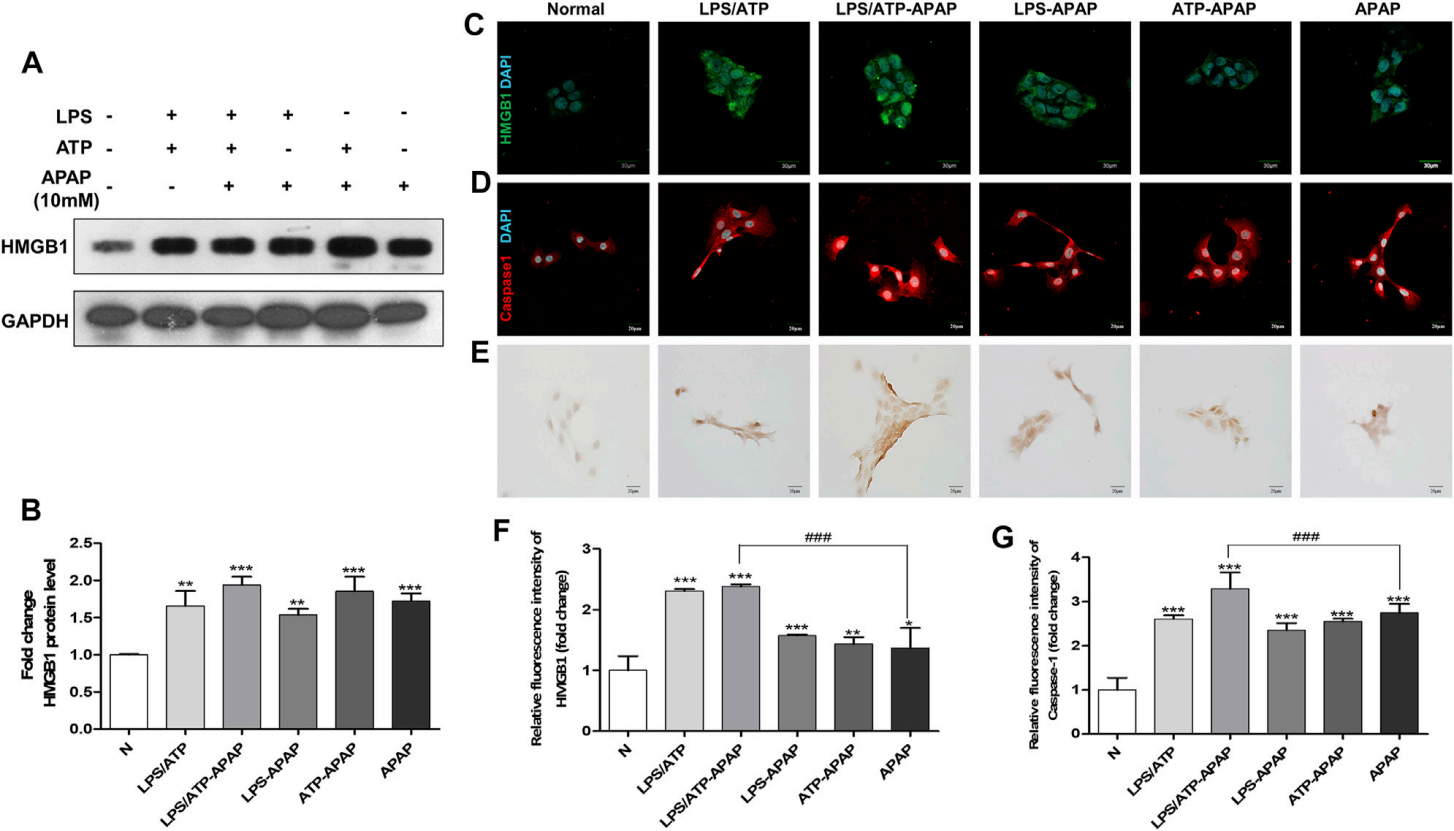
Changes in inflammatory indexes in vitro model of APAP induced by LPS/ATP combined with stimulation. **(A,B)** HMGB1 protein expression and quantitative analysis relative to GAPDH. **(C,D)** Immunofluorescence staining of HMGB1 and Caspase-1, the image is magnified 600×. **(E)** TUNEL staining, the image is magnified 400×. **(F,G)** Relative fluorescence quantitative analysis of C and D. ^#^ *p* < 0.05, ^##^ *p* < 0.01, ^###^ *p* < 0.001, significantly different from APAP group. *p < 0.05, **p < 0.01, ***p < 0.001, significantly different from the normal group. One-way ANOVA followed by Tukey’s test. The data are expressed as the mean ± SD of five independent assays with at least three replicates.

In order to better simulate the actual situation of inflammation *in vivo*, ATP combined to LPS was treated to APAP-stimulated hepatocyte as a second signal to magnify inflammasome-related inflammation. The expression of HMGB1 in cells increased to a higher level after APAP combined with LPS and ATP (Figures 2A–C,F). The results of Caspase-1 showed the same trend as that of HMGB1 (Figure 2D,G), indicating that the second signal formed by LPS/ATP combined with APAP was related to the activation of Caspase-1. In addition, the degree of apoptosis further increased (Figure 2E).

### Neutrophil Extracellular Traps Shaped the Crosstalk Between Neutrophils and Hepatocytes During Acetaminophen Stimulation

**FIGURE 3:**
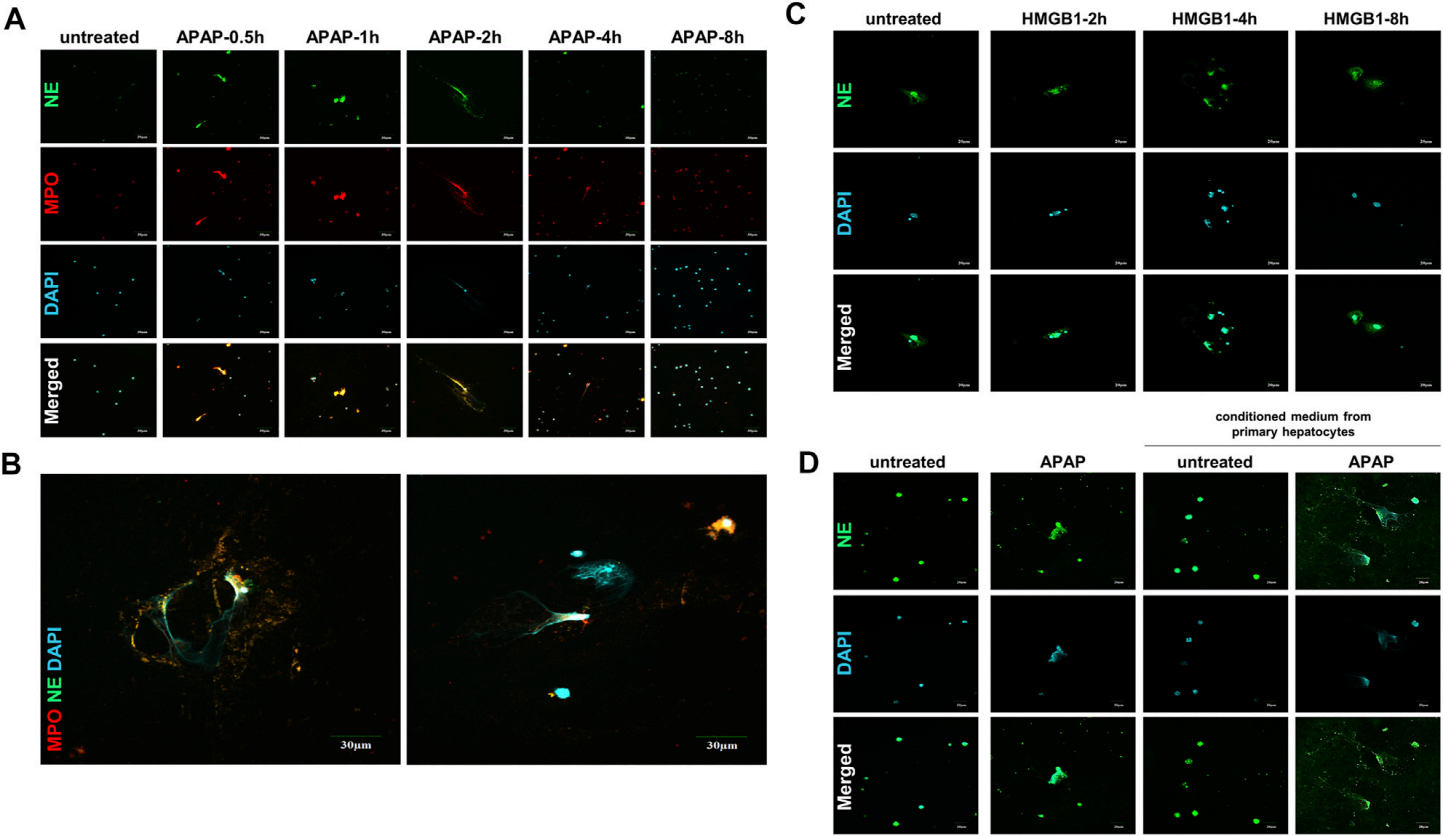
APAP induces the formation of primary neutrophils NETs and there is a cross-talk between HMGB1 mediated neutrophils and APAP-induced primary hepatocytes. To study the effect of APAP on the formation of neutrophil NETs, APAP (5 mM) treated AML12 cells for 0–8 h **(A,B)** The parallel results of NE and MPO immunofluorescence staining and APAP treatment for 2 h. **(C)** Neutrophils treated with 100 ng/ml HMGB1 protein for 0–8 h. **(D)** Crosstalk between primary mouse liver cells and neutrophils. After the primary mouse hepatocytes was treated with APAP (5 mM) for 24 h, the supernatant was used as a conditioned medium and cultured on neutrophils for another 2 h. The results of the immunofluorescence of NE in the neutrophils were obtained. The display image is the result of 600× magnifications taken by Olympus confocal microscope.

The formation of NETs are considered to be a kind of cell death with the result of inflammation (Papayannopoulos, 2018; Wang et al., 2020). The expression of NE and MPO, the landmark indicators of NETs activation, increased within 8 h of APAP treatment in mouse primary neutrophils, and reached the peak within 2 h, when part of the NETs formed completely, and then this phenomenon gradually disappeared (Figures 3A,B).

When the liver is damaged and produces inflammation, neutrophils are first recruited to the liver, which is the first line of defense against aseptic inflammation (Villanueva et al., 2011; Khandpur et al., 2013). We studied the possible role of hepatocytes on the recruitment of neutrophils to the liver after APAP stimulation under aseptic inflammation, and to explore the cross-dialogue between primary hepatocytes and neutrophils stimulated by APAP. First, the HMGB1 protein (100 ng/ml) was processed on the neutrophils under a time gradient of 0–8 h. In the nuclear staining, it can be seen that NETs gradually formed and the expression of NE increased (Figure 3C). Subsequently, the primary mouse hepatocytes were stimulated with APAP for 24 h, and the culture medium was treated on neutrophils for 2 h. Compared with APAP directly stimulating neutrophils, it was found that the damaged hepatocytes caused the neutrophils to be further activated, NETs formation increases, and NE expression increases (Figure 3D). The above data showed that the crosstalk between neutrophils and primary hepatocytes may be mediated by HMGB1.

### Fasting Deteriorated Autophagy and Inflammatory Neutrophil Extracellular Traps Formation in Acetaminophen-Induced Mice Liver Injury

**FIGURE 4:**
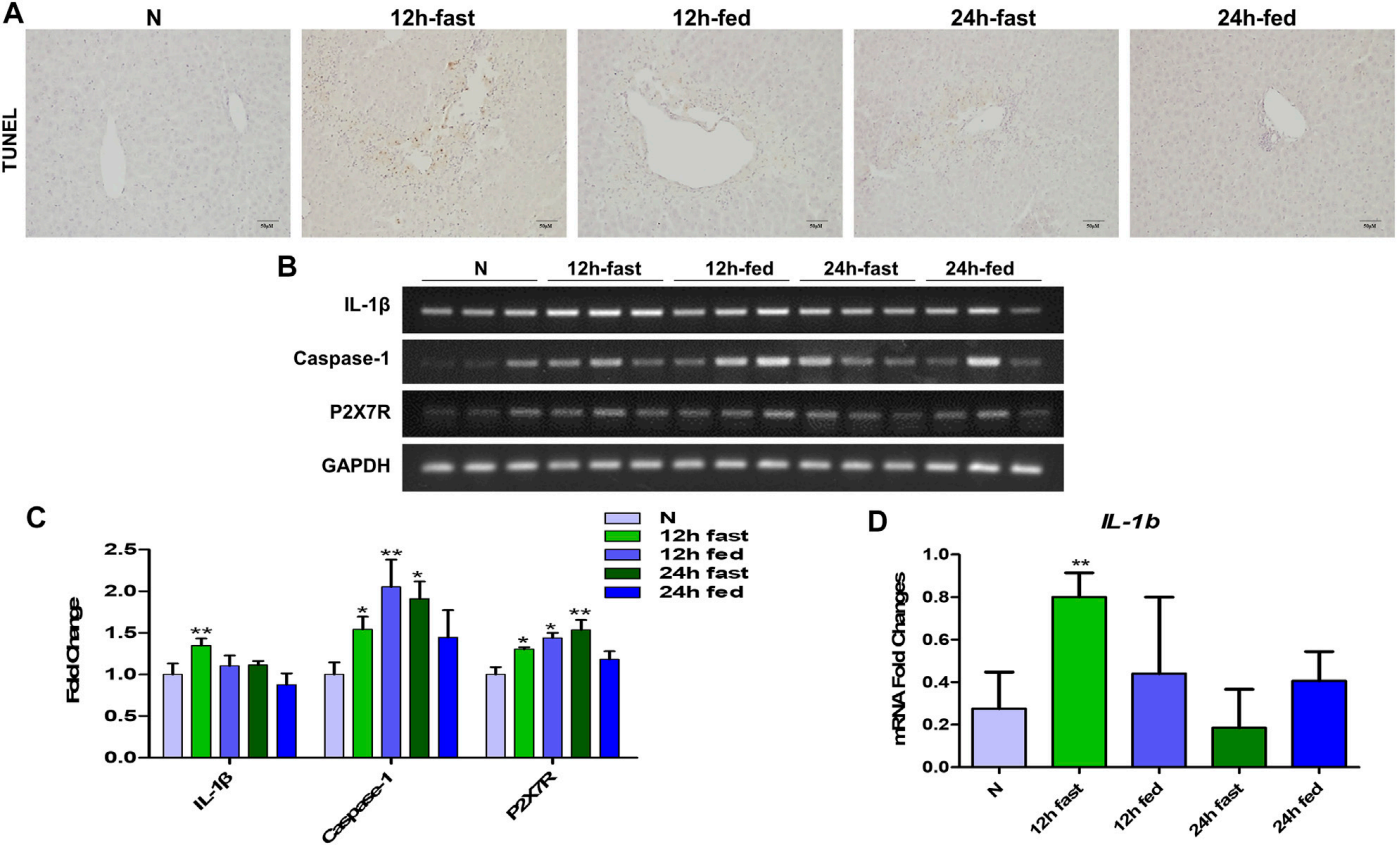
Effect of fasting factors on the *in vivo* model of liver injury induced by APAP. C57B/6 mice were randomly divided into five groups: normal group, APAP (300 mg/kg) 12 h fasting or not fasting group and 24 h fasting or not fasting group. **(A)** TUNEL staining, the image was magnified by 200×. **(B,C)** mRNA expression of IL-1β, Caspase-1 and P2X7R and their relative expression levels relative to GAPDH. **(D)** (E) Real-time quantitative PCR results of IL-1β. *p < 0.05, **p < 0.01, ***p < 0.001, significantly different from the normal group. One-way ANOVA followed by Tukey’s test. The data are expressed as the mean ± SD of five independent assays with at least three replicates.

According to the preliminary experiment, it was found that whether mice fasted or not before APAP stimulation had a significant effect on the degree of APAP-induced liver injury in mice. Therefore, in order to eliminate the interference of fasting factors in subsequent *in vivo* experiments, we verified the effects of fasting factors on APAP liver injury and inflammation. Before APAP 12 and 24 h treatments, we fasted mice for 12 h but not to refrain from water. After TUNEL staining, it was found that the fasting mice treated with APAP showed more severe liver apoptosis (Figure 4A) than the non-fasting mice, and the mRNA levels of inflammatory indexes IL-1β, Caspase-1 and P2X7R were significantly increased (Figures 4B–D). This shows that the *in vivo* model of APAP mice is sensitive to fasting factors.

**FIGURE 5:**
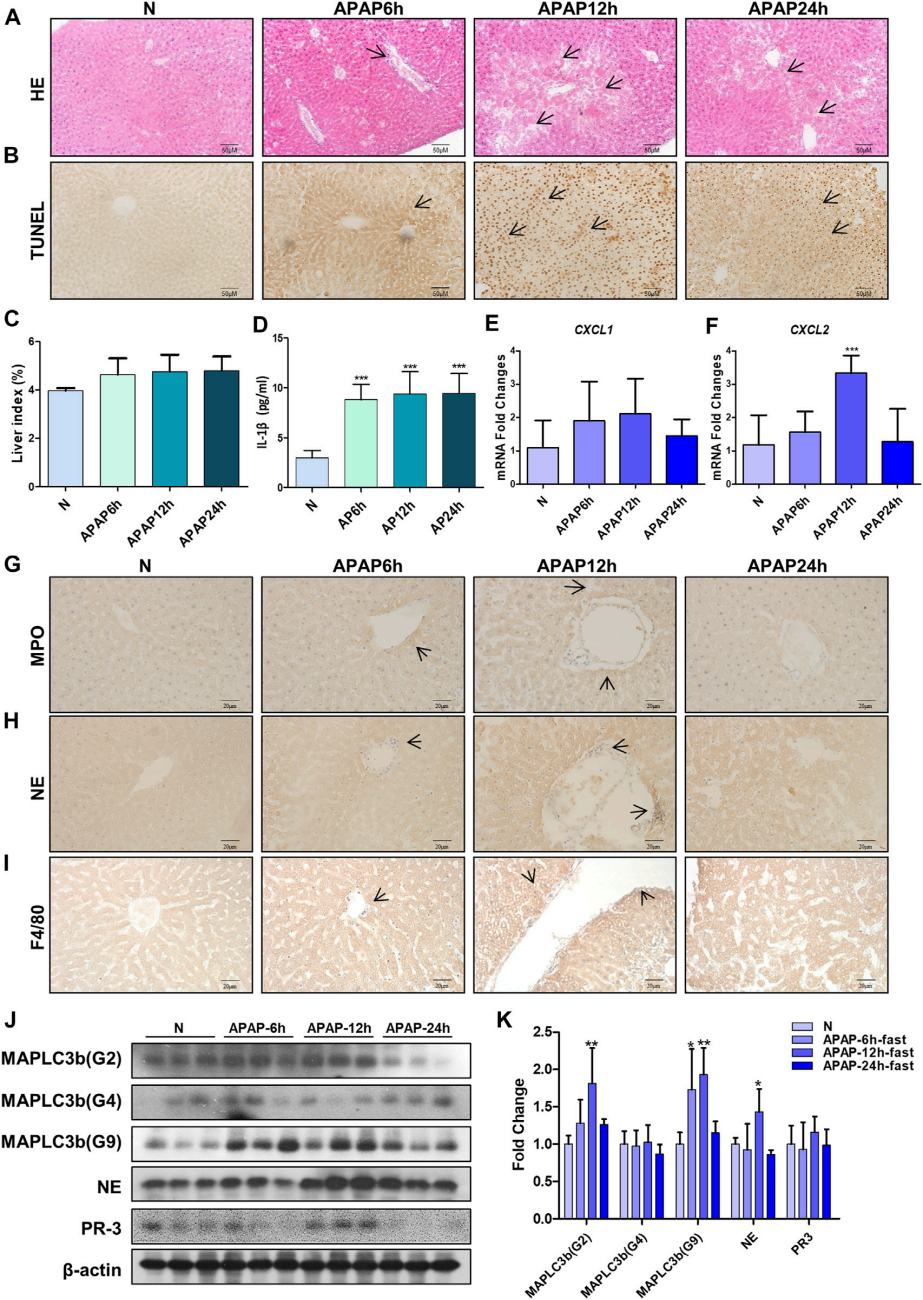
Evaluation of liver injury induced by different time gradients of APAP under fasting condition and the expression of chemokines and autophagy-related proteins in the *in vivo* model of APAP liver injury. C57BL/6 mice were randomly divided into normal groups, APAP (300 mg/kg) 6, 12 and 24 h groups. Fasting began 12 h before APAP was given. **(A,B)** HE and TUNEL staining, the image is magnified 200×. Specific staining indicated by arrow. **(C)** Liver weight index. **(D)** Expression of IL-1β in serum. **(E,F)** Real-time quantitative PCR results of chemokine CXCL-1 and CXCL-2. **(G–I)** Immunochemical staining of MPO, NE and F4/80, the image is magnified 400×. **(J,K)** MAPLC3β (Gmur2), MAPLC3β (Gmur4), MAPLC3β (Gmur9), NE and PR3 protein expression and gray-scale density analysis relative to β-actin. *p < 0.05, **p < 0.01, ***p < 0.001, significantly different from the normal group. One-way ANOVA followed by Tukey’s test. The data are expressed as the mean ± SD of five independent assays with at least three replicates.

In order to study the mechanism of liver injury induced by APAP under fasting, we examined the immune response in the liver of mice at different times. It was found that under the condition of fasting for 6, 12, and 24 h, the arrangement regularity of hepatocytes decreased, the cellular stroma enlarged with a large number of lymphocytes infiltration, the apoptosis of mouse tissue increased and the liver index increased (Figures 5A–C). The level of IL-1β in serum of mice increased significantly from 6 h and remained stable after 6 h (Figure 5D). During the production of NETs, the mRNA expression of chemokines CXCL1 and CXCL2 produced by neutrophils (Figures 5E,F), characteristic proteins PR3 and MPO, NE and macrophage marker F4/80 protein expression levels were the highest at 12 h (Figures 5G–K). Autophagy is the stress response produced when the liver is damaged. The expression of autophagy-related proteins MAPLC3β (G-2) and MAPLC3β (G-9) increased at 12 h, and MAPLC3β (G-4) did not change (Figures 5J,K). It is shown that the process of liver damage caused by APAP under fasting conditions is related to the process of autophagy.

**FIGURE 6:**
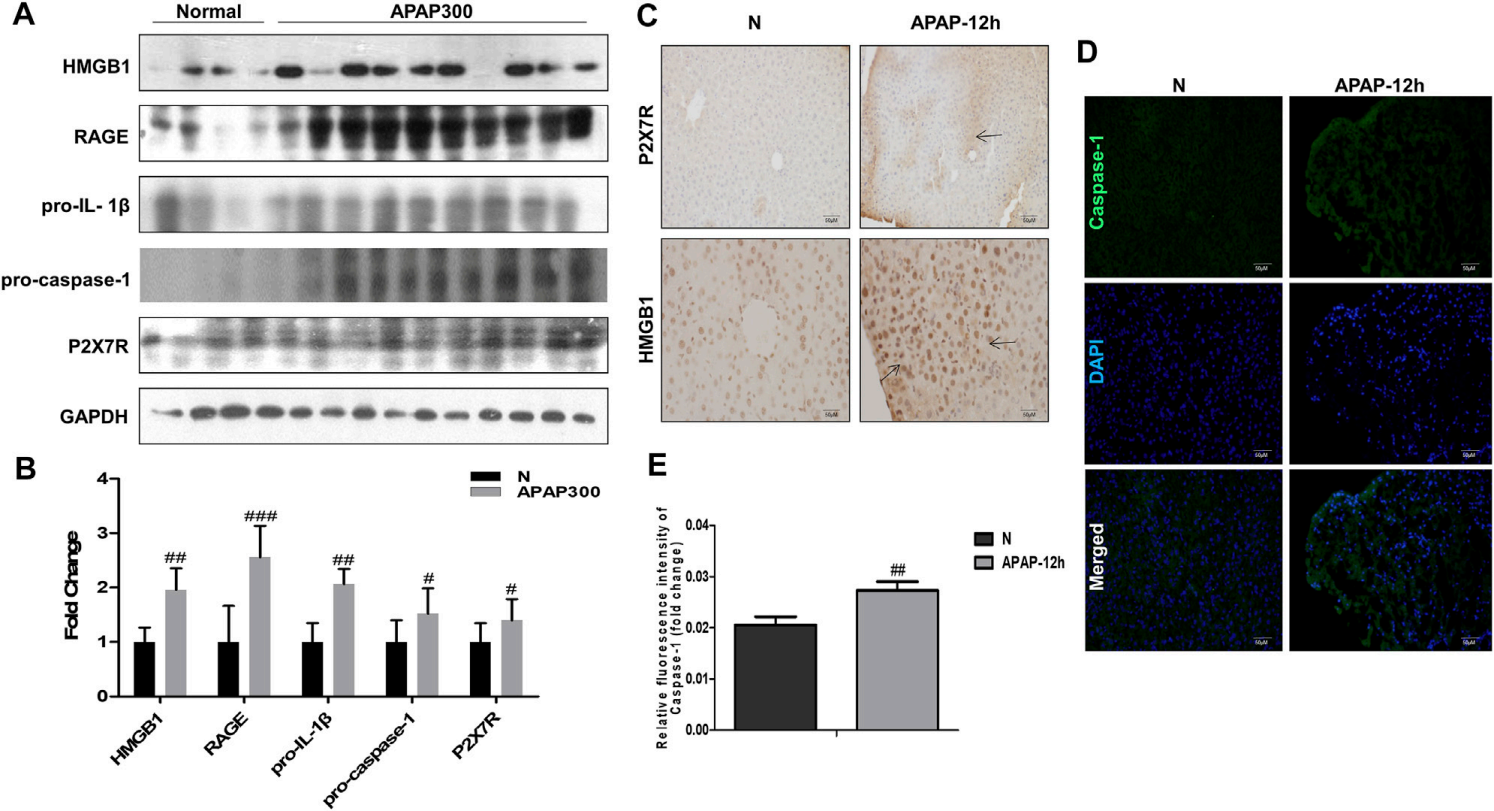
The inflammatory mechanism of liver injury in APAP fasting model was observed at 12 h in the damage phase. **(A,B)** Normal group and fasting group, 12 h after APAP (300 mg/kg) treatment, western blot results of HMGB1, its receptor proteins RAGE, IL-1β, Caspase-1 and P2X7R and its quantitative analysis relative to GAPDH. **(C)** HMGB1 and P2X7R immunohistochemical staining, image magnification 200×, of which HMGB1 magnification 400×. The position indicated by the arrow is the characteristic stain. **(D,E)** FLICA staining and relative fluorescence quantitative analysis of Caspase-1, the image is magnified 200×. ^#^ *p* < 0.05, ^##^ *p* < 0.01, ^###^ *p* < 0.001, significantly different from normal group. One-way ANOVA followed by Tukey’s test. The data are expressed as the mean ± SD of five independent assays with at least three replicates.

Results of (Figure 6) showed that there was a strong inflammatory reaction in the phase of liver injury induced by APAP under fasting condition, and the expression levels of HMGB1, RAGE, pro-IL-1β, P2X7R and pro-caspase-1 were markedly increased. Among them, P2X7R, as the receptor of ATP, can activate the NLRP3 inflammasome, complete the release of Caspase-1, and participate in the body’s inflammatory response. This indicates during APAP injury, the increase of HMGB1 and IL-1β may amplify the inflammatory response through the activation of Caspase-1 by RAGE and P2X7R.

### Neutrophils Are Indispensable in Acetaminophen-Induce Liver Injury

**FIGURE 7:**
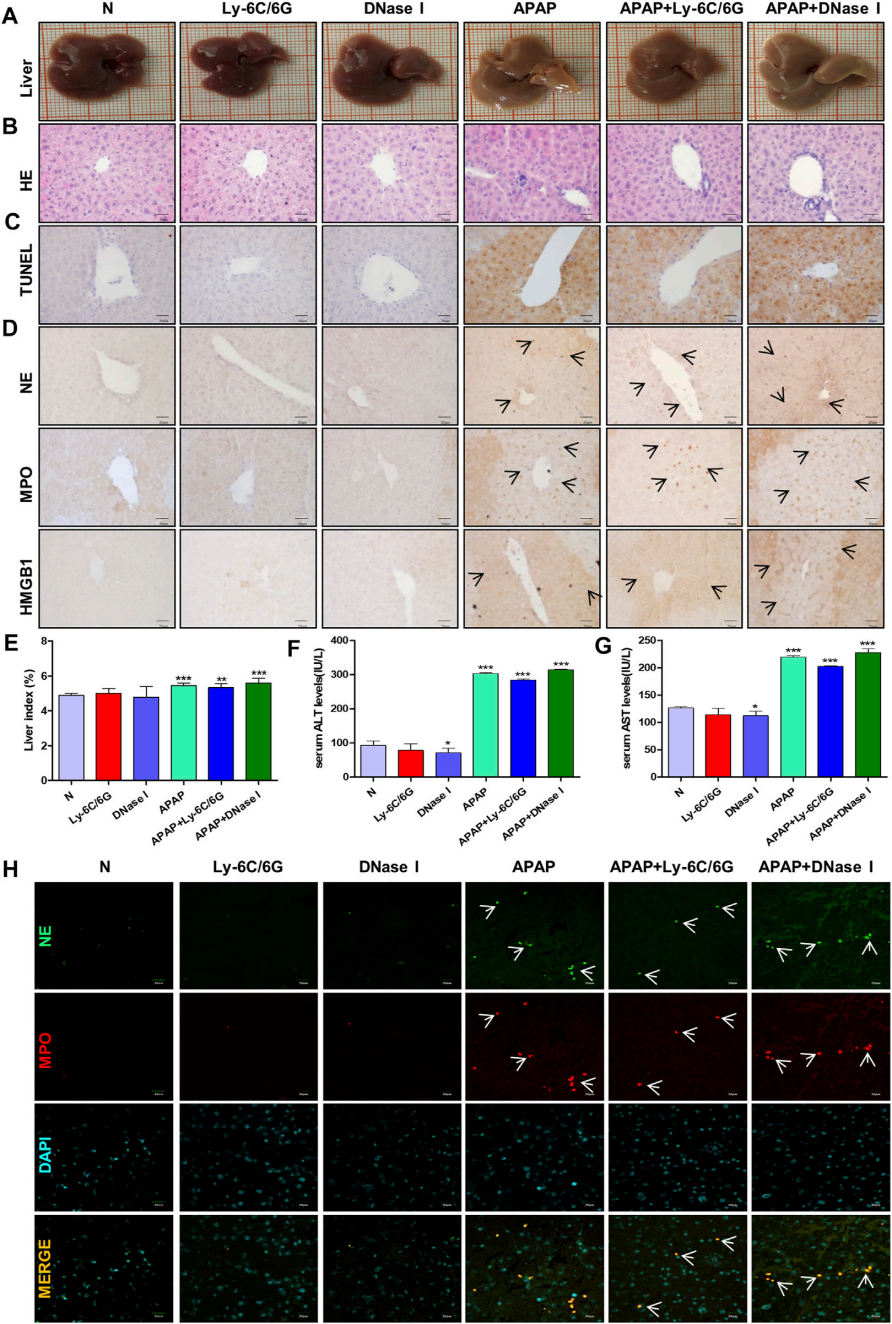
Effects of neutrophils and NETs on liver injury induced by APAP. C57BL/6 mice were randomly divided into six groups: normal group, Ly6C/6G group, DNase I group, APAP group, APAP + Ly6C/6G group and APAP + DNase I group. Ly6C/6G (200 μg/mouse) was injected intraperitoneally 24 h before APAP (300 mg/kg) treatment. DNase I (10 mg/kg) was injected intraperitoneally 1 h after APAP treatment. **(A)** Morphology of mouse liver tissue samples. **(B,C)** HE and TUNEL staining. **(D)** Immunohistochemical staining of NE, MPO and HMGB1. **(E)** Liver weight index. **(F,G)** The level of ALT and AST in serum. **(H)** Immunofluorescence staining of frozen sections of NE and MPO. Green and red fluorescent label NE and MPO, orange yellow label NE and MPO dual expression. All images were magnified by 400×. Specific staining indicated by arrow. *p < 0.05, **p < 0.01, ***p < 0.001, significantly different from the normal group. One-way ANOVA followed by Tukey’s test. The data are expressed as the mean ± SD of five independent assays with at least three replicates.

Neutrophils are the most abundant effector cells in the human immune system. NETs formed after infection exists for several days and can be degraded by nuclease DNase I in the cytoplasm (Wong et al., 2015; Han et al., 2019). In order to study the role of neutrophils in APAP-induced liver injury, we established a neutrophil depletion model and carried out enzymatic hydrolysis of NETs produced by neutrophils during immune behavior. Compared with the normal group, there was no significant change in the liver morphology of mice treated with Ly-6C/6G and DNase I alone, but the liver tissue surface color uniformity, elasticity and smoothness of the group treated with APAP were significantly reduced, and the tissue staining showed Severe inflammatory infiltration, tissue damage and apoptosis (Figures 7A–C), NE, MPO and HMGB1 expression significantly increased (Figure 7D), liver index, ALT and AST also significantly increased (Figures 7E–G). At the same time, by immunofluorescence double staining of NE and MPO, a clear NETs reaction was observed, indicating that NETs participated in the injury response during the liver injury process (Figure 7H).

### *Chikusetsusaponin V* Suppressed HMGB1 Release and Caspase-1 Activation in Hepatocytes and Neutrophil Extracellular Traps Formation in Neutrophils

**FIGURE 8:**
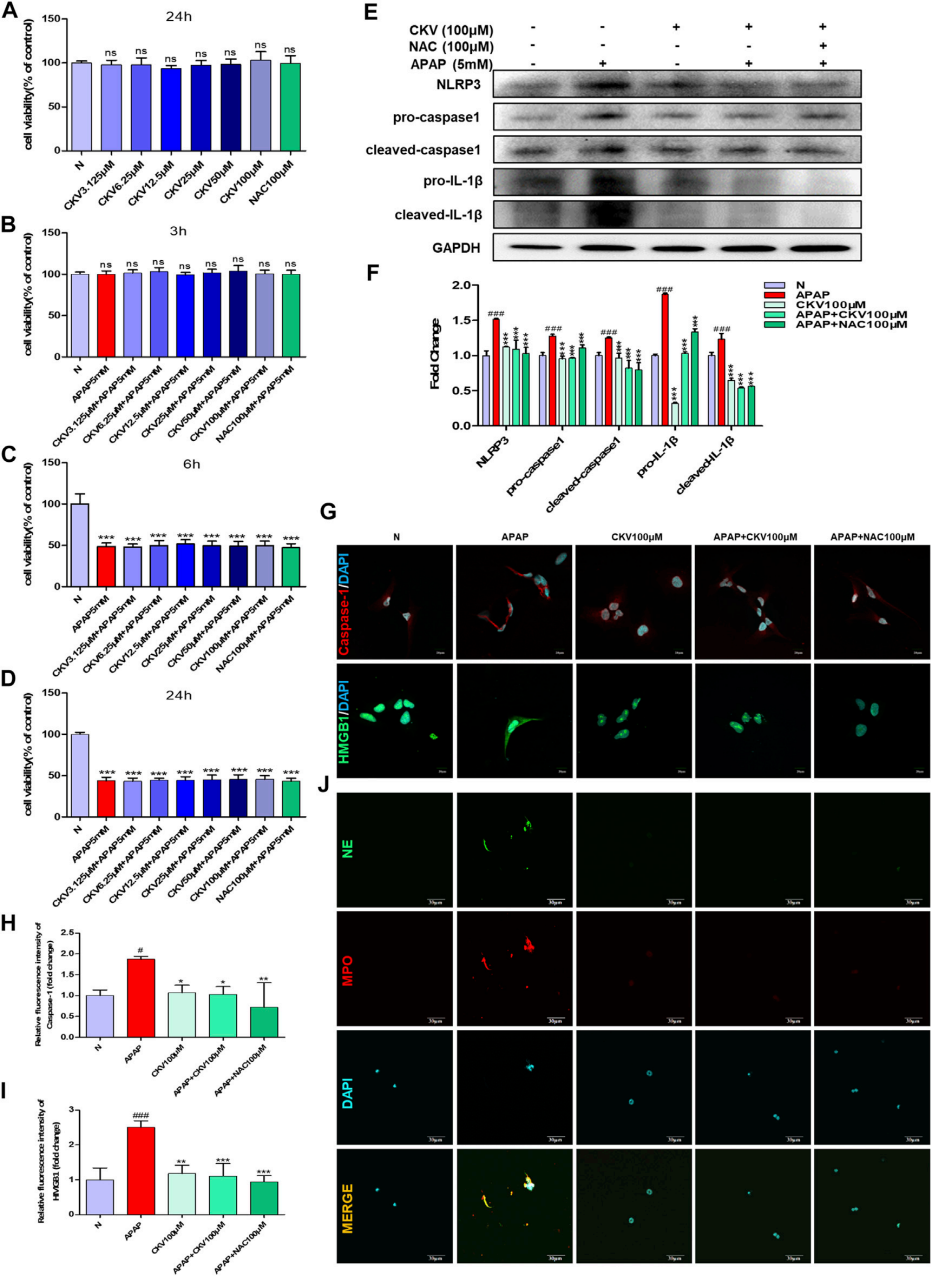
CKV reduces liver damage by inhibiting the release of HMGB1 and the activation of Caspase-1 in liver cells after APAP stimulation, and the formation of neutrophil NETs. **(A)** 24 h survival rate of AML12 cells stimulated by 3.125–100 μM CKV and 100 μM NAC. **(B–D)** 3.125–100 μM CKV and 100 μM NAC and APAP (5 mM) combined to stimulate the survival rate of AML12 cells at 3, 6 and 24 h **(E,F)** AML12 cells were simultaneously treated with 100 μM CKV and APAP (5 mM) for 3 h to detect the protein expression and relative quantitative analysis of NLRP3, pro-caspase1, cleaved-caspase1, pro-IL-1β and cleaved-IL-1β. **(G–I)** Immunofluorescence staining was used to detect the expression and relative quantitative analysis of Caspase-1 and HMGB1. The red fluorescent label is Caspase-1 and the green fluorescent label is HMGB1. **(J)** NE and MPO immunofluorescence staining. Green fluorescence labeled NE, red fluorescence labeled MPO, orange yellow labeled double expression of NE and MPO. All images were magnified by 600×. ^#^ *p* < 0.05, ^##^ *p* < 0.01, ^###^ *p* < 0.001, significantly different from normal group; *p < 0.05, **p < 0.01, ***p < 0.001, significantly different from APAP group. One-way ANOVA followed by Tukey’s test. The data are expressed as the mean ± SD of five independent assays with at least three replicates.

CKV and NAC didn’t show any toxicity within 100 μM in AML12 cells during 24 h (Figure 8A). After that, the effect of CKV with a concentration gradient of 3.125–100 μM on the survival rate of AML12 cells stimulated by APAP (5 mM) for 3, 6 and 24 h was examined. The results showed that APAP did not produce obvious toxicity to AML12 cells at 3 h, but significantly at 6 and 24 h (Figures 8B–D). The results of *in vitro* experiments showed that the treatment of, CKV (100 μM) significantly decreased the expression of NLRP3, Caspase-1, IL-1β, and HMGB1 in AML12 cells induced by APAP, and inhibited NETs formation in APAP-treated primary neutrophils with decreasing NE and MPO (Figures 8E–J).

### *Chikusetsusaponin V* Alleviated Acetaminophen-Induced Inflammatory Response in Mice via Inhibiting Caspase-1-Mediated HMGB1 Release and Neutrophil Extracellular Traps Formation

**FIGURE 9:**
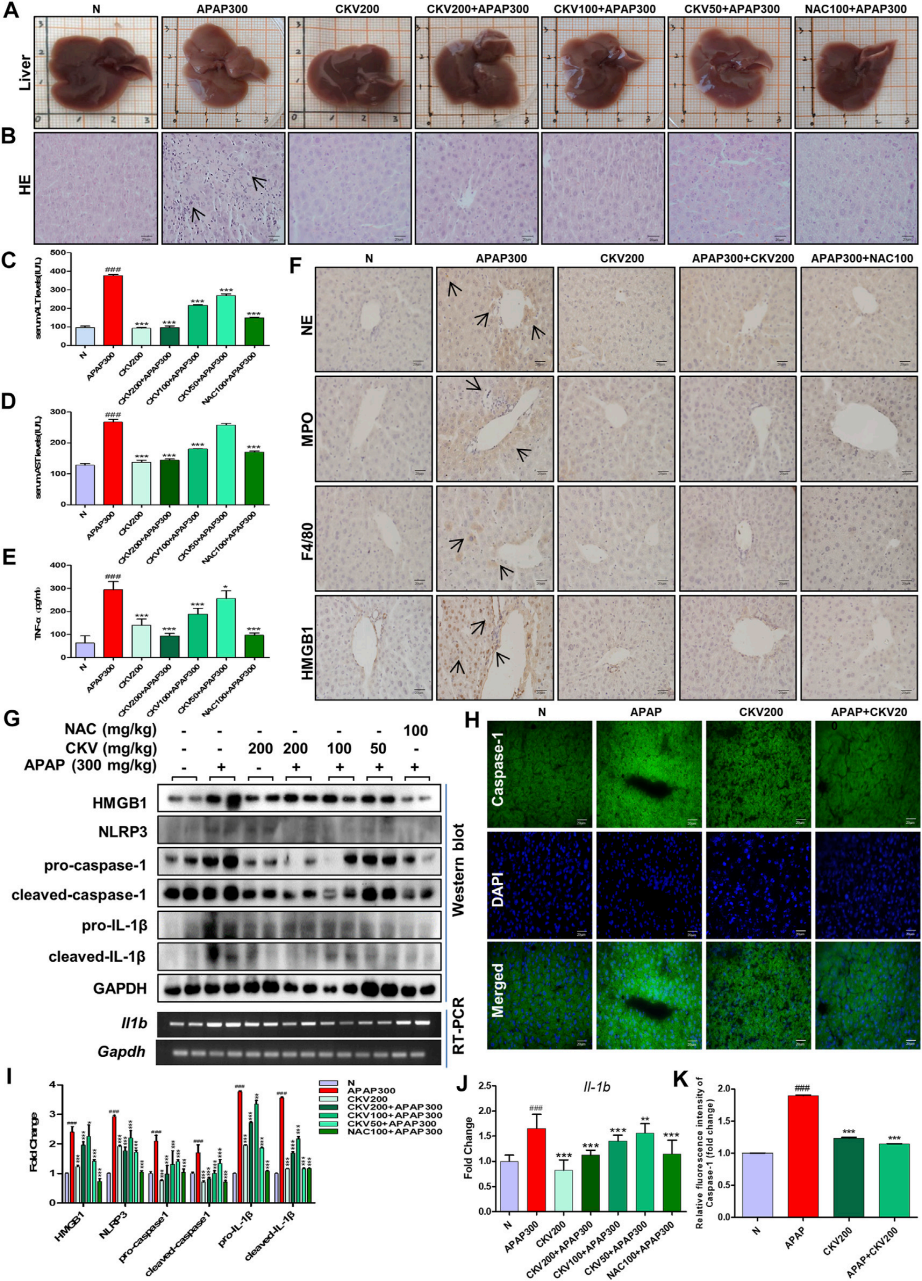
Effect of CKV on liver injury and inflammation induced by APAP in mice. C57BL/6 mice were randomly divided into normal group, APAP alone group, APAP combined with CKV (200 mg/kg), CKV (100 mg/kg) or CKV (50 mg/kg) group, positive drug NAC (100 mg/kg) control group, and CKV (200 mg/kg) alone treatment group. CKV was given 1 h before APAP treatment, and mice were sacrificed 12 h after APAP treatment to collect samples. **(A)** Morphology of mouse liver tissue samples. **(B)** HE staining. **(C,D)** The level of ALT and AST in serum. **(E)** The ELISA results of different concentrations of CKV on the expression of TNF- α in serum of mice induced by APAP. **(F)** Immunohistochemical staining of NE, MPO, F4/80 and HMGB1. **(G)** The protein expression of HMGB1, NLRP3, pro-caspase1, cleaved-caspase1, pro-IL-1β, and cleaved-IL-1β and the mRNA expression level of IL-1β. **(H)** FLICA staining of Caspase-1. **(I,J)** G relative to GAPDH gray-scale density and gene expression analysis. **(K)** H relative fluorescence quantitative analysis. All images were magnified by 400×. The position indicated by the arrow is the characteristic stain. ^#^ *p* < 0.05, ^##^ *p* < 0.01, ^###^ *p* < 0.001, significantly different from normal group; *p < 0.05, **p < 0.01, ***p < 0.001, significantly different from APAP group. One-way ANOVA followed by Tukey’s test. The data are expressed as the mean ± SD of five independent assays with at least three replicates.

Based on the previous experimental results, we detected the intervention effect of CKV on liver injury induced by APAP *in vivo*. APAP (300 mg/kg) was used to simulate the injury phase of liver poisoning caused by excessive APAP under fasting for 12 h. The dosage of CKV was set to 200 mg/kg, 100 mg/kg and 50 mg/kg, and NAC (100 mg/kg) was used as the positive control drug in animal experiment. The results showed that the three doses of CKV, high, medium and low, can alleviate the liver damage caused by APAP overdose. It can improve the uniformity, elasticity and smoothness of the liver tissue surface of mice (Figure 9A). HE staining revealed that the regularity of liver cell arrangement caused by APAP injury was reduced, and the intercellular substance became larger with a large amount of lymphocyte infiltration. CKV treatment can significantly improve the above characteristics (Figure 9B). In addition, ALT and AST also significantly decreased (Figures 9C,D). At the same time, CKV reduced the expression of TNF-α (Tumor Necrosis Factor-α) in the serum of mice caused by APAP (Figure 9E). The administration of CKV (200 mg/kg) also has an intervening effect on the infiltration of immune cells in the process of liver injury caused by APAP, effectively reducing the recruitment of neutrophils, monocytes and macrophages, manifested as NE, MPO, HMGB1 And F4/80 expression decreased (Figure 9F). CKV can significantly inhibit the expression level of HMGB1, NLRP3, Caspase1 and IL-1β in the liver at a dose of 200 mg/kg-50 mg/kg, and the effect is the best at a dose of 200 mg/kg. (Figures 9G–K).

## Discussion

There is growing evidence that inflammation aggravates the pathological development of a variety of acute and chronic liver diseases (Tilg and Moschen, 2010; Silva and Peixoto, 2018). However, the role of inflammatory response in APAP-induced liver injury is not clear, and there is still much controversy (Woolbright and Jaeschke, 2015). In particular, under the current global situation of dealing with the epidemic situation of COVID-19, elucidating the mechanism of inflammation in the pathological process of APAP acute liver poisoning will not only find a new therapeutic target for the development of antidotes, but also provide reference strategies for the treatment of clinical drug-induced liver poisoning, which is of great significance.

Compared with other species, the acute hepatotoxicity model of APAP in mice best reproduced the pathological damage of human liver (McGill et al., 2012; Xie et al., 2014). In the process of APAP *in vivo* modeling, species, sex and genetic background are the key factors affecting modeling. Compared with mice, rats are not sensitive to the severity of hepatotoxicity caused by APAP, and the correlation with human clinical condition is very limited. In addition, although there was no significant difference in the severity of APAP-induced hepatotoxicity in humans, the sensitivity of mice to APAP liver injury was affected by gender factors. The level of glutathione in female mice was higher than that in males. Glutathione can be detoxified by binding to N-Acetyl-P-Benzoquinonimine (NAPQI), a toxic intermediate metabolite of APAP, so female mice are not comfortable and evaluation of APAP liver injury model *in vivo* (Du et al., 2014). In addition, some mouse models cannot fully simulate human APAP-induced acute liver failure, such as C3Heb/FeJ mice, after excessive administration of APAP, the death of mice is due to massive bleeding in the liver resulting in hypovolemic shock (Du et al., 2014; Liang et al., 2018), while human drug-induced liver failure is usually caused by sepsis. Although mice have been widely recognized as an experimental animal model for the study of APAP liver injury, it is worth noting that there are many key factors involved in mechanism research in different strains of mice due to differences in genetic background, including genetic response to acute inflammatory stress or differences in the activity of liver metabolic enzymes (Seok et al., 2013). Similarly, *in vitro* studies cannot fully reflect the metabolism of APAP *in vivo*. Therefore, strict and controllable clinical toxicity trials are very important to find therapeutic strategies for APAP liver poisoning in natural products. In this study, an *in vitro* and *in vivo* model of APAP hepatotoxicity was established to study the mechanism of the inflammatory reaction.

The cytotoxicity results showed that HepG2 cells showed higher tolerance to APAP (Figures 1A–C) than AML12 cells at the same concentration of APAP. Therefore, HepG2 as an *in vitro* model of APAP is still open to question, so in the follow-up experiments, we chose AML12 cells as the main object of study. APAP has obvious phase in the process of hepatotoxicity. In this study, when establishing *in vivo* and *in vitro* models, we focused on the mechanism of inflammation in the damage phase and the effect of APAP on the pathological development of the inflammatory reaction. The inflammatory reaction of mouse liver induced by APAP developed from 6 to 24 h after administration, and entered the stage of repair and regeneration after 24 h (Jaeschke et al., 2012). According to the experimental results *in vivo*, it was found in the pre-experiment that fasting factors caused mice to be more sensitive to APAP liver damage, and the level of inflammation in fasting mice was higher and more significant. When mice were treated with APAP 12 h, the release of inflammatory cytokines IL-1β in serum and liver, and the mRNA expression of inflammation-related protein Caspase-1, P2X7R and chemokines CXCL1 and CXCL2 were significantly increased. 12 h may be the peak of hepatic inflammatory response induced by APAP, which can be used as a time point for the study of inflammatory mechanism. Therefore, fasting and phase are important factors to be considered in the study of APAP liver injury model, especially in the study of inflammation-related mechanisms (Figures 4–6). In addition, we found that the expression of autophagy-related proteins MAPLC3β increased significantly during this process. Since autophagy is a stress response when the liver is damaged and destabilized, it can degrade cell debris and recycle organelles through lysosomes. But autophagy is not just about removing metabolic waste, it has also been reported to be involved in the development of some diseases. This shows that autophagy may be involved in the peak phase of the inflammatory response induced under APAP fasting conditions (Figure 5).

The essence of inflammatory reaction is the reflection of the body’s confrontation with various inflammatory factors. More and more evidence shows that inflammation aggravates the pathological development of a variety of acute and chronic liver diseases. After APAP enters the damage phase, the DAMPs produced by cell death is the key node to induce inflammatory response. At present, as a typical danger signal, HMGB1 is a hot topic in the research of inflammatory mechanism. Therefore, we chose HMGB1 as the starting point to study the downstream mechanism of APAP-induced liver inflammation. In the *in vitro* experiment, the protein expression of HMGB1 began to increase after administration of APAP 1 h, and reached the peak at 3 h. After that, although the expression of Caspase-1 decreased somewhat, it still maintained a high level compared with the normal group, and this process was accompanied by the activation of Caspase-1 (Figures 1E–J). Then, the change trend of HMGB1 expression after APAP stimulation was verified again by LPS/ATP co-stimulation. The results showed that after LPS/ATP combined with APAP stimulation, the expression of HMGB1 increased significantly, still accompanied by the activation of Caspase-1 (Figure 2). In the *in vivo* experiment, the correlation between HMGB1 and Caspase-1 (Figure 6) was still observed in the 12 h damage phase when the inflammatory reaction was at its peak. Therefore, it is concluded that the response of liver inflammation induced by APAP may be mediated by HMGB1, which depends on the activation of Caspase-1, which is an important finding of this study. Because the index of HMGB1 appears in the early stage of inflammation *in vivo*, it can be used as an indicator for the diagnosis of APAP excess, but this still needs to consider the observability of the actual level *in vivo* and the interaction with other cells.

As an important inflammatory factor, IL-1β plays an important role in mediating inflammatory response in a variety of liver disease models. Some studies have found that the release level of IL-1β actually produced in mice with hepatotoxicity is very low, IL-1β receptor deficient mice do not have a protective effect on APAP, and inflammation cannot establish a relationship with the metabolic activation of APAP (Williams et al., 2010; Hoque et al., 2012). In particular, there was no increase in IL-1β in patients with excessive APAP in clinical trials, and there was no significant difference between patients who survived and died (Williams et al., 2014). This study also makes a preliminary study on the focus of the debate on IL-1β. According to the results of *in vivo* studies, the expression of IL-1β increased continuously during 6–24 h after APAP treatment (Figure 5D). However, in the *in vitro* experiment, an interesting phenomenon was found by ELISA results (Figure 1D). With the passage of time, the release of IL-1β changed from negative correlation to positive correlation, and remained flat at 6 h. However, 6 h is often the dividing point at which APAP develops from metabolic phase to damage phase. From this point, it is inferred that the change of IL-1β expression in inflammation induced by APAP, like APAP, may also have a temporal nature, which inspires us to explore the phase more deeply. However, the role of IL-1β in the inflammation of APAP hepatotoxicity is still in doubt, and it is necessary to carefully consider it as a therapeutic target for liver inflammation caused by APAP.

Innate immunity plays an important role in coping with aseptic inflammation caused by liver damage. In humans and mice, APAP-induced liver cell death releases a large amount of DAMPs, including HMGB1, nuclear DNA fragments and mitochondrial DNA, which makes macrophages produce pro-inflammatory mediators, leading to early neutrophils and monocytes cells are recruited to the liver, and there is now a general consensus (Jaeschke et al., 2012). In order to study the possible effect of the endogenous signal HMGB1 released by cell death of hepatocytes on neutrophils recruited to the liver after APAP stimulation, we explored the cross-talk between primary mouse liver cells and neutrophils under APAP stimulation. The results showed that both the supernatant of damaged liver cells after APAP stimulation and the direct administration of HMGB1 caused neutrophils to be further activated and produce higher levels of NE, indicating that in APAP-induced liver injury, neutrophils The cross-talk with primary hepatocytes may be mediated by HMGB1 (Figures 3C,D). At the same time, in the *in vivo* model of neutrophil depletion, by neutralizing neutrophils with Ly-6C/6G antibody, APAP-induced liver damage was reduced to a certain extent, and the expression of HMGB1 was reduced (Figure 7). The above shows that after APAP stimulation, the activation of neutrophils may be related to the HMGB1 released by hepatocyte death, and the activated neutrophils may aggravate liver damage.

In addition to phagocytosis and degranulation, the recruited neutrophils also trigger ROS (Reactive Oxygen Species) production, activate MPO and NE, and form NETs. HMGB1 released after cell death will also accelerate the formation of NETs by neutrophils and release a variety of pro-inflammatory mediators. *In vitro* experiments show that APAP can directly promote the release of NETs from neutrophils. APAP is rapidly activated to form a network structure within 8 h of stimulation, reaching its apex within 2 h, and the expression of neutrophil activation markers NE and MPO increases, and then gradually disappeared (Figures 3A,B). At the same time, in the *in vivo* model of DNase I degradation of NETs, we found that liver damage did not alleviate, but instead produced more HMGB1 protein. This shows that DNase I can only eliminate the DNA grid that produces NETs, but cannot affect the release of chemokines and inflammatory factors during the activation of neutrophils (Figure 7). Therefore, the formation of NETs is a kind of cell death with inflammatory consequences. Inhibiting the production of NETs can reduce liver inflammation caused by APAP. When APAP is at a safe dose, it enters the liver through blood circulation and enters the metabolic phase, less than 10% of APAP is metabolized by CYP2E1 (Cytochrome P4502E1) to produce toxic metabolites NAPQI, which is under the catalysis of glutathione sulfur transferase, combines with glutathione to generate non-toxic mercapturic acid, which is excreted from the body through the urinary system. However, after excessive APAP, CYP2E1 metabolism will produce a large amount of NAPQI, which will quickly deplete the glutathione pool, resulting in the synthesis of a large number of cell protein conjugates, especially mitochondrial protein conjugates, and then initiate the injury phase. In the injury phase of APAP liver poisoning, the hallmark pathological phenomenon is the oxidative stress in the organelles caused by mitochondrial protein conjugates. The downstream event of mitochondrial oxidative stress caused by APAP is an inflammatory response that occurs when the cell ruptures and releases DAMPs after cell death. Although NAC is the only effective drug for the treatment of liver damage caused by APAP so far, its therapeutic window is relatively narrow and only effective within 12 h after overdose. However, research on extracting natural products from plants has revealed a large number of promising molecules that can be used to develop antidote against APAP liver toxicity. Under the study of the mechanism of APAP-induced liver injury, we gave natural product CKV treatment and found that CKV can not only regulate the formation of neutrophil NETs, but also inhibit the release of cell death endogenous molecule HMGB1 and the activation of Caspase-1. Thereby reducing neutrophil recruitment and mitochondrial oxidative stress-induced liver inflammation, and play an active role in APAP-induced liver injury (Figures 8, 9).

**FIGURE 10:**
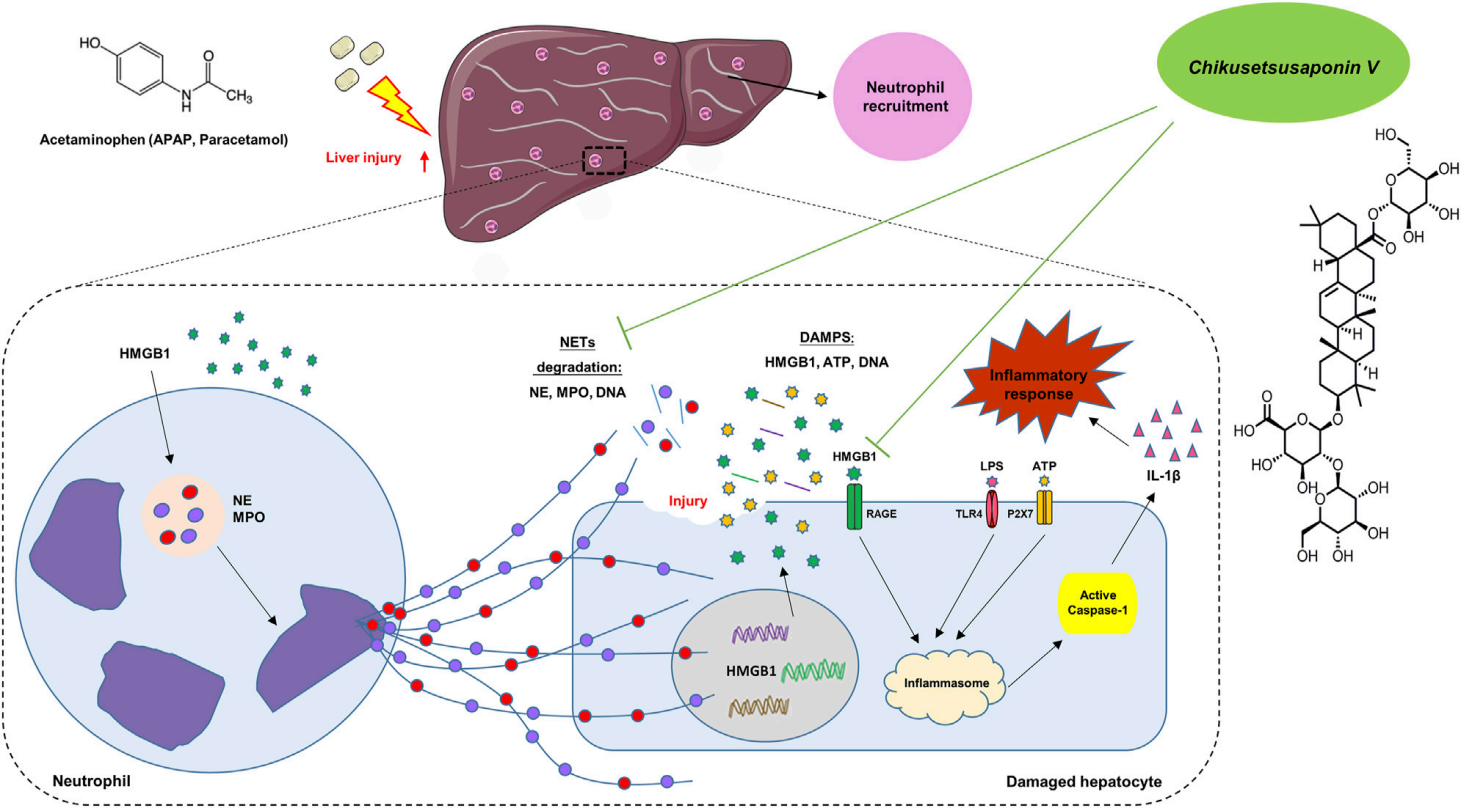
Summary of the underlying mechanism of CKV on alleviating APAP-induced hepatic injury.

In conclusion, in the early stage of APAP injury, neutrophils and NETs formation participated to transmit the inflammatory response between hepatocytes and neutrophils via Caspase-1-dependent HMGB1 release. CKV possesses a positive intervention to NETs formation and Caspase-1-HMGB1 mediated hepatocytes damage (Figure 10), suggesting CKV might be a promising candidate for drug-induced liver injury.

## Data Availability

The raw data supporting the conclusions of this article will be made available by the authors, without undue reservation.

## Abbreviations

APAP, acetaminophen; ATP, adenosine triphosphate; CKV, chikusetsusaponin V; CXCL1, C-X-C motif chemokine 1; CXCL2, C-X-C motif chemokine 2; CYP2E1, cytochrome P450 2E1; DAMPs, damage associated molecular patterns; HMGB1, high-mobility group box 1 protein; IL-1β, interleukin-1β; LPS, lipopolysaccharide; MAPK, mitogen activated protein kinase; MPO, myeloperoxidase; NAPQI, N-acetyl-P-benzoquinonimine; NE, neutrophil elastase; NETs, neutrophil extracellular traps; NF-κB, nuclear factor-κB; NLRP3, NOD like receptors protein P3; RAGE, receptor for advanced glycation endproducts; ROS, reactive oxygen species; TLR4, toll-like receptor 4; TNF-α, tumor necrosis factor-α.
